# Perceptions, relationships, expectations, and challenges: Views of communication and research for scientific dissemination in Brazilian Federal Institutes

**DOI:** 10.1371/journal.pone.0258492

**Published:** 2021-10-14

**Authors:** Tássia Galvão, Priscilla Rayanne e Silva Noll, Matias Noll

**Affiliations:** 1 Department of Education, Instituto Federal Goiano, Ceres, Goiás, Brazil; 2 Faculdade de Medicina, Universidade de São Paulo (FMUSP), Universidade de São Paulo, São Paulo, Brazil; 3 Physical Education Department, Universidade Federal de Goiás, Goiânia, Goiás, Brazil; 4 Department of Sports Science and Clinical Biomechanics, University of Southern Denmark, Odense, Denmark; Universitat Luzern, SWITZERLAND

## Abstract

Communicating Brazilian science still seems to be a challenge for journalists and researchers of public institutions of education and science. In this sense, this research aims to identify and analyze scientists’ perceptions regarding the work of journalists, the relationship between these groups, the expectations, and the challenges of science communication in two Federal Institutes of Education in Brazil. We conducted a mixed study in the qualitative stage with the participation of 30 interviewees, and in the quantitative stage, journalists and researchers answered a questionnaire (n = 242). Our results indicated that the work of science communication is not carried out properly in both Institutes and that there is a lack of articulated work among both journalists, communicators, and researchers. The relationship between these groups needs to be built jointly. In this respect, the biggest challenges are to institutionalize science communication, establish a science communication plan, and overcome internal relationship barriers. Our results may underpin science communication policies and policies for scientific dissemination both institutional or even national levels.

## Introduction

Science communication is understood as making research and science processes and results public in a language that can be understood by a greater number of lay people. It has been evolving and changing over time. The term has different meanings across the world—in the United States, it is understood as scientific communication, but also as a meaning that encompasses the science communication to peers and the lay citizen [1]. In China, the term popularization of science is used to refer to dissemination actions, events, and initiatives aimed at society in general [2]. In United Kingdom, more recently, the term public engagement of science has been conceived from the perspective of public communication in various sectors [2].

In Brazil, there is a differentiation between “science communication” and “scientific communication” [3]. The first term refers to publicization among those who are not part of the scientific environment, and the second refers to communication between specialists [4]. Despite the different conceptions, in several countries, both terms have similar purposes. In this context, communication professionals and researchers are the main protagonists in the work of scientific dissemination. This occurs mainly in the institutions and centers where scientific knowledge is produced. However, relationships are not always easy. The relationship between journalists and scientists should aim to integrate the institution, its researchers, and communities through interaction and systems for exchanging messages and communication [5] for the promotion of science, which still represents a major challenge for public institutions and their audiences. These participatory and collaborative dynamics need to be adopted for the encouragement, motivation, and commitment of the actors in the process of making and communicating science [6].

In this context, we are facing two distinct fields of action: scientific culture and the culture of communication [7]. Each of them has specificities and particularities that are often not clear, thus creating barriers in this relationship. Despite this, these two poles need to connect, mainly because communication professionals are relevant for scientific communication. Among the activities to be carried out by them are selecting and publishing information on different channels according to journalistic criteria, sharing messages, paying attention to regulations, and thus, being effective in their science communication process [8,9].

Furthermore, it is necessary to establish a continuous dialogue between researchers and journalists, whose challenges in relating to people often comes from their own lack of knowledge about people from different groups [10]. In addition to the work they do, this unknown environment may create uncertainties in the relationships between communication professionals and researchers. This is also due to historical contexts, the dynamics of institutions, and the lack of science communication practice [11]. Another barrier related to this context is the fragile institutional support to the development of this integration and interaction between professionals from different areas [12].

The institutional environment also does not focus on stimulating and promoting science communication activities carried out with diligence and priority by the communicators or their researchers. In this context, the institutionalization process in this field is established with the creation of norms that may, in a certain way, provoke a change of internal behavior in the organization [13]. The articulation and carrying out of debates and actions for scientific dissemination along with communication campaigns may contribute to this process of behavioral change and the creation of a culture of science communication, stimulating the creation of identity among groups and expectations of benefits, and personal and professional satisfaction [14].

In this regard, it must be considered that today, more than ever, actions are needed to promote dialogue between the institution, the researcher, and the communities in search of a connection that is still less explored by scientists and those responsible for public decisions [15]. Among the ways pointed out by the National Academies of Sciences, Engineering [9], forming partnerships and developing a research dissemination agenda are worthy examples. In relation to this reality inserted in institutional contexts, we realize that most studies only analyze the relationship between the communication sectors and the media or scientists and the press, and do not indulge in the internal analyzes of organizations [16,17]. This research attempts to conduct such an analysis as media and the impact of the press on the formation of public opinion have long been researched.

In this perspective, other studies point out the need to carry out planning in communication, treating this communication as strategic in the name of science in addition to establishing clear objectives of dissemination [18,19]. One of the problems highlighted by Besley et al. [19] is that organizational communication is still concerned more with contributing to the organization or institution and less with promoting scientific knowledge, given that the organizations themselves do not prioritize the advancement of science. Specifically on the relationship between scientists and communication professionals in organizations, Koivumäki and Wilkinson [20] emphasize that negative impacts on the quality and cohesion of scientific dissemination may occur due to the need to establish an identity and purposes between these two groups. Furthermore, Rödder [21] places this field of discussion—science communication in organizations—as an emerging field in the conceptual perspective for an “organizational sociology of scientific dissemination.”

The present study is placed in these educational environments and spaces in the context of public Brazilian teaching and research institutions. As a locus of investigation, we selected two Federal Institutes of Education, Science, and Technology (FIs—*Institutos Federais de Educação*, *Ciência e Tecnologia*) that are part of a Network with more than 650 units distributed throughout Brazil [22]: the Federal Network for Professional, Scientific, and Technological Education (RFEPCT—*Rede Federal de Educação Profissional*, *Científica e Tecnológica*), created in 2008 [23]. The Network has more than 1 million students [22] pursuing courses ranging from medium technical level to graduation and post-graduation. Annually, more than two thousand researches are developed by high school and university students through the scientific initiation programs in these institutions [24]. In this context, we highlight the following questions: What are the perceptions of researchers, journalists, and communicators regarding the work of disseminating researches? How is the relationship between journalists and researchers?

Given this and the attempt to build a cooperative dialogue between journalists and scientists and, thus, to develop an effective science communication, this article aims to identify and analyze the researchers’ perceptions regarding the work of journalists and communicators, the relationship between these groups, the expectations, and the challenges of scientific dissemination in two Brazilian FIs. Based on our study, public science communication policies and policies for scientific dissemination may be proposed at the institutional or even national level. This means that the results may bring benefits to the communities, especially those related to the researched institutions. This happens through the effective communication of scientific processes, either by the benefits of the research results, or by its direct impact on society, whose results will become known due to the work to be carried out by the social communication sector.

## Method

### Nature and type of research

This research uses the mixed approach, integrating qualitative and quantitative data collection methods [25–27] through an institutional case study.

### Research context and subjects

**Fig 1:**
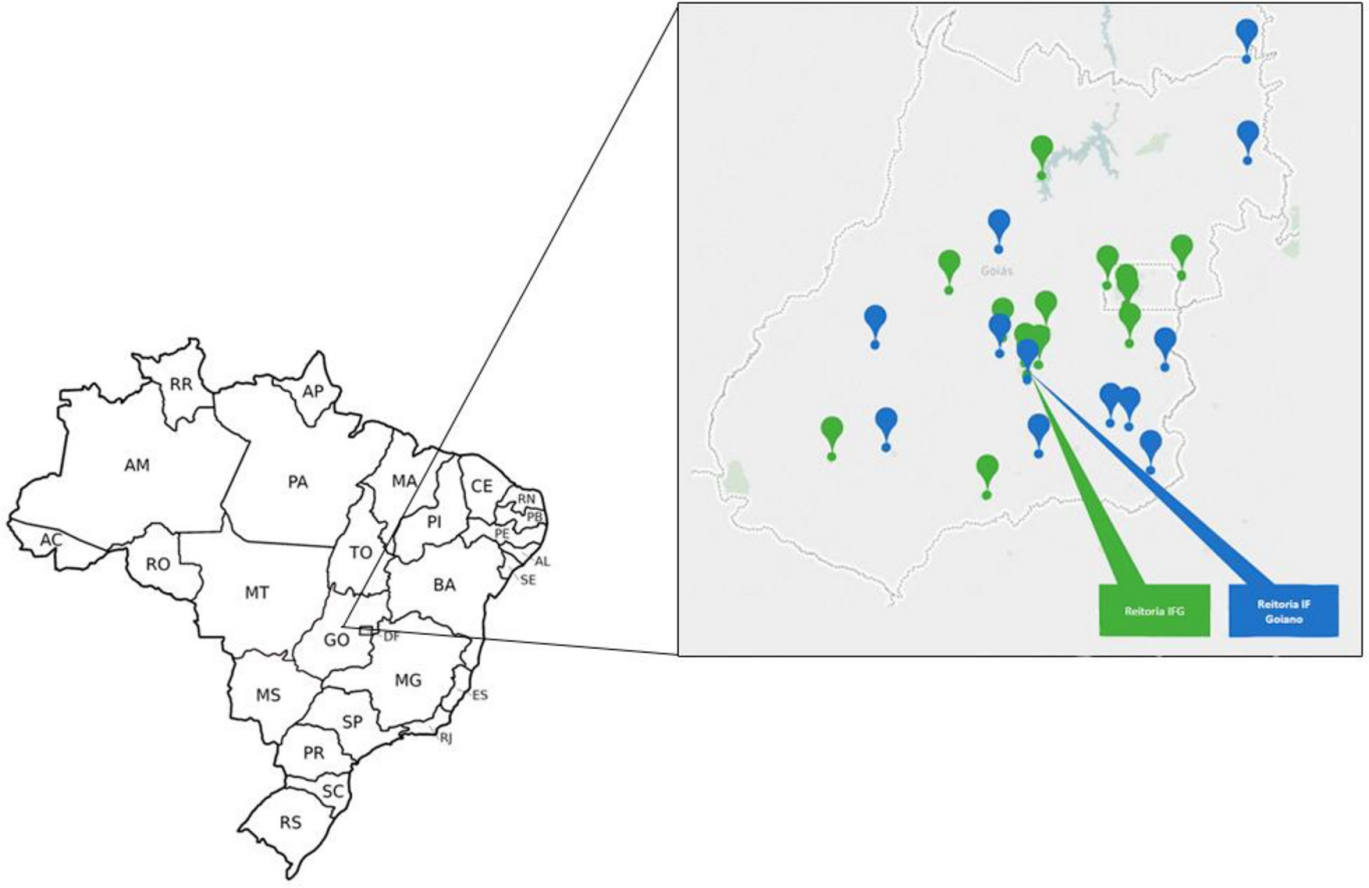
Map of Brazil with emphasis on the Federal Institutes of the State of Goiás, in the Central region of the country. Note. The map of Goiás state was built by the authors based on the Reordering Plan of the units of the Federal Network. The green dots represent the IFG campuses and the blue ones, the IF Goiano.

The study was carried out at the management campus (administrative campus) and at two more campuses of two RFEPCT institutions (Fig 1): The Federal Institute of Goiás (FIG) and Federal Goiano Institute (IF Goiano). Both are in Goiás, in central Brazil. The management campus, from each FI, is located in the state capital, in Goiânia, and are the macro administrative units.

FIG academic community is constituted by 16,564 students and 2,204 civil professionals, including educators and technical-administrative staff [22]. On the other hand, the IF Goiano is constituted by 18,658 students and 1,975 educators and technical-administrative staff [22]. This information is from 2019 year.

Professionals from the communication and research sectors, researchers from institutional programs for scientific initiation and technological development registered in the years 2018 and 2019, and managers participated in this study. The objective was to map the evaluations and opinions tracing convergences and divergences in the perceptions of each group. The subjects participating in the research (Table 1) are part of a universe from 14 FIG and 13 IF Goiano campuses: Journalists and Communicators (JC); Research Managers (RM); Communication Managers (CM); Research Deans (RD–called also as Pro-rectors of Research); and researchers (educators and students) from scientific initiation and technological development programs. The student researchers’ group were both scholarship or volunteer at programs registered in the last 2018 and 2019.

**Table 1:** Subjects participating in the research.

Groups	Population	Qualitative Sample	Quantitative Sample			
	(n)	(n)	(n)	Female	Age [years] (Mean ± SD)	Experience [years] (Mean ± SD)
Journalist and Communicator (JC)	50	5	23	52.2%	35.7 ± 6.9	5.7 ± 2.2 ^a^
Communication Manager (CM)	2	2	---	---	---	---
Research Manager (RM)	31	5	11	45.5%	38.4 ± 9.1	5.8 ± 6.7 ^a^
Research Dean (RD)	2	2	---	---	---	---
Student Researcher (StuR) *	700	8	52	57.7%	21.4 ± 4.9	3.2 ± 1.7 ^b^
Research Supervisors (ReS) *	619	8	156	41%	40.5 ± 8.1	8.1 ± 5.6 ^a^

*Researchers who are part of the projects registered in 2018 and 2019 of the scientific initiation and technological development programs/FIG and IF Goiano.

^a^ Experience working at the Institute.

^b^ Experience studying at the Institute.

The subjects participated voluntarily in the research; whose ethical precepts were followed according to the Brazilian legislation. The research was approved by the IF Goiano (n° 08501319.0.0000.0036) and FIG research Ethics Committees (n° 08501319.0.3001.8082).

### Data collection procedures

In this study, four data collection procedures were used: a) documentary analysis, b) structured interview, c) questionnaire with closed questions, and d) analysis of news coverage. The procedure for validating the questionnaires and interviews is presented ahead.

#### a) Documents

The corpus comprised the main documents that govern the functioning and establish the guidelines of the two FIs: the Institutional Development Plan and the Institutional Pedagogical Project, updated in 2018. Moreover, minutes of Research, Innovation and Postgraduate Policies, and the Regulation of the Scientific Initiation and Technological Development Program were used. This was the first stage of data collection and took place in August 2019.

#### b) Interview

The interviews, guided by a structured questionnaire, were recorded using a voice recorder and performed individually at each location. This stage took place between the months of November 2019 and February 2020. The interviewees were identified in their statements according to the acronyms in Table 1 and numbered according to the chronological order of the interviews to guarantee the anonymity of the participants. Before starting the interview, each of them received two copies of the Informed Consent Form, which was read and signed by the participant. Fully interview guide, specific for each group, can be accessed in the Supplementary material 1 in S1 File.

Among the themes of the interviews were the subjects’ perceptions regarding the work of scientific dissemination, with a focus on science journalism, carried out in the two FIs; the challenges faced in the process of publicizing science, expectations of how to overcome these challenges; and the evaluation of the relationship between journalists and communicators, and researchers. The responses constitute—together with quantitative research and news analysis—an overview of the work carried out in the institutions in addition to self-assessments and knowledge regarding the roles that each segment must play.

#### c) Survey questionnaire

The questionnaire was designed with 23 closed and three open questions. The first five were designed to characterize the sample of participants. From the full questionnaire, this study considered five closed questions about: 1) the agreement or disagreement in relation to the interaction of communication professionals with researchers, 2) the accessibility of communication professionals, 3) satisfaction with the dissemination of research, 4) the degree of knowledge about the role of the journalist and communicator as the only ones who can do scientific dissemination, and 5) the researcher’s role as a scientific disseminator.

The questionnaire was directed to the group: JC, RM, Student Researcher (StuR), and Research Supervisors (ReS). The survey was sent electronically between September 2019 and February 2020 in an online form using Google Forms. This instrument was developed following a five-point Likert scale: 1 = Strongly disagree, 2 = Disagree, 3 = Undecided, 4 = Agree, and 5 = Strongly agree. For data analysis, the following points were grouped: “Strongly disagree” with “Disagree” and “Strongly agree” with “Agree”, such that the expected frequencies were greater than 5 [28], thus ensuring reliability of the instrument.

#### d) Analysis of news coverage

The 100 news items published in the institutional portals of FIG and IF Goiano were collected for coverage analysis. The aim was to illustrate and complement the quantitative and qualitative data with the journalistic practice of scientific themes from the two institutions. Data collection was carried out on October 10, 2019, and the last 100 articles published were counted. At the FIG and IF Goiano, the materials collected refer to the period from June 24 to October 10 and June 28 to October 10, respectively.

In addition to quantifying the articles published on the main websites (ifg.edu.br and ifgoiano.edu.br), the structures of themes and content were also analyzed. We investigated whether, during the analyzed period, news were published regarding research projects—in progress or their results—and how the content was represented in the texts. Further, we compared this production with the interview statements and quantitative data to detect intersections, disagreements, or discrepancies and, thus, make sense of the analyzes through comparison [29].

#### e) Validation of research instruments

Questionnaires and interviews were subjected to content validation before the study started. For the analysis of the questions, 50 external participants (not included in the analysis from the present study) including researchers, journalists, and students were invited to evaluate the preliminary version of the instruments. The validation was performed according to the recommendations [30,31]. They establish the appropriate validation procedures, minimum quantity, and level of education of the specialists. The following criteria were adopted individually in each item: 1) organization, 2) objectivity, 3) clarity, 4) ease of reading, 5) understanding of the content, and 6) pertinence. These were presented in the introduction of the validation questionnaire with definitions.

The questions were assessed according to the Content Validation Index (CVI), which measures—in percentage—the agreement of judges regarding the aspects and items of the instrument [31]. Initially, each item (question) was analyzed individually and later, the full instrument. A four-point Likert scale was used (1 = not relevant, 2 = item needs major revision, 3 = item needs minor revision, and 4 = relevant item).

First, the evaluators responded to the survey and subsequently made the evaluations. To find out if, in a global way, the instrument contemplated the objectives of the research, the judges evaluated with yes or no answers. For each item evaluated, the CVI was calculated by dividing the number of responses 3 and 4 by the total number of responses to the questionnaire. Accepted items had CVI ≥ 75% [31]. Only one item was excluded from the quantitative research, of which the CVI was equal to 70% in all criteria including the pertinence of the question. In the same instrument, one more item was reformulated. Despite having obtained a CVI of 50% in the criteria of clarity and objectivity, it was considered an important issue with CVI = 100% in the pertinence criterion. After applying the instruments, we proceeded to the analysis of qualitative and quantitative data, as will be described below.

### Data analysis

The description of the data analysis for this study is presented in four sections: document analysis, analysis of the interviews, analysis of the news, and finally, analysis of the questionnaire.

#### Documents

Institutional titles were analyzed using content analysis, according to Amado [32] and Bardin [33], based on the presence and number of occurrences or absence of the terms, scientific dissemination and science journalism. After the first assessment of the number of meanings, we proceeded to the synthesis of meanings and the senses in which these words are highlighted in the documents, detecting the titles and situations in which they are referenced. So, we were able to recognize the institutional reality, official or not, in which scientific dissemination was carried out in the two institutions.

#### Interviews

The data collected in the interviews were transcribed in full to perform the content analysis, according to the classification of stages as evidenced by Franco [29], Amado [32] and Bardin [33], which includes pre-analysis, material exploration, and treatment of results. The conduct of the interviews enabled the interaction between the researcher and the subjects—a characteristic of dialogue and bilateral communication, which is fundamental in qualitative research—in addition to deepening the perceptions of researchers and journalists regarding the reality of scientific dissemination [34–37]. From the transcribed interviews, we established semantically the thematic categories of analysis (categorization) a posteriori, composed of the context and registration units and the indicators [32,33], contemplating the key ideas of this corpus.

In the first step, we listed the common elements in the analysis by group to search for patterns or connections between the categories and the indicators [38]. In a second step, we compared the results by categories and groups to analyze the connections, intersections, and discrepancies. These results, when combined with the analysis of quantitative research and news coverage, allowed us to triangulate the findings to develop a more solid basis for the factors associated with the objectives of this study [32,39].

Among the categorized themes, we considered those related to situation definition codes, such as those that refer to the perceptions regarding the work of the communication sector in the dissemination of research; those of the subjects’ perspectives, which include suggestions for improvements, advances, and development of the work of science communication; those of relationship and social structure, with the analysis of the relationship between journalists and researchers; and those of context, with the enumeration of challenges. Finally, the strategy codes were considered, with suggestions for overcoming these challenges [38].

#### News analysis

The 100 articles published on the institutional websites were collected, and their titles were inserted in an electronic spreadsheet in the Excel software. A specific file was prepared for each institution, with three columns: the title of the subject, theme, and first paragraph of the text (only for the subjects related to the research projects). The themes were categorized after reading the titles and all the articles, as it was not possible to identify the subject in some of them just by reading the headline (headline is a term used in journalism, which means the prominent title of the story). Subsequently, we collected the full articles on the websites that referred to the research projects.

In the next step, we counted the news by thematic category and established the subcategories only in matters related to institutional research to identify communication priorities within the themes of scientific research. Finally, we analyzed the textual structure, according to the precepts of journalism and scientific dissemination materials, according to Gomes [40] and Queiroz & Becker [41]. We established the previous criteria to be analyzed in each subject: subject and area, nature of the sources consulted, need for prior knowledge for understanding, adequacy to the public, focus on science journalism or scientific dissemination, meeting the criteria of news [42], language resources [43,44], visual aspects, and integration with social networks.

#### Questionnaires

For the analysis of the questionnaires, the answers to the closed questions were coded and tabulated, and statistical data analysis was performed using the SPSS 26.0 software and descriptive statistics. We identified the frequency in relation to disagreement or agreement with the following items: a) interaction between journalists and researchers, b) accessibility of communication professionals, c) if the scientific research carried out at the institution is properly disseminated, d) the role of journalists and communicators in science communication, and e) the role of researchers in science communication.

## Results

The results are subdivided into the following sections: a) scientific dissemination in institutional documents; b) the insertion of research in institutional portals; c) characterization of the research subjects; d) perceptions, relationships, and challenges; and e) scenario of scientific dissemination and the roles of each actor in this context.

### a) The science communication in institutional documents

Among the official documents analyzed, both the terms—science communication and science journalism—were not detected in the FIG norms in any of the listed titles: Institutional Development Plan, Institutional Pedagogical Project, minutes of Research, Innovation and Postgraduate Policies, and the Regulation of the Scientific Initiation and Technological Development Program. This means that the searched terms were not inserted directly. In the documents of the IF Goiano, two occurrences of the term “scientific dissemination” were found in the Institutional Development Plan and Institutional Pedagogical Project. Despite this, they are limited to the purposes and characteristics of the programs to be developed by the Library System. Therefore, they have no connection with the media. The term “science journalism” was not detected in any of the documents.

### b) The insertion of research in institutional portals

The mapping of 100 news items published on the institutional websites of the analyzed institutions shows a reduced circulation of journalistic articles about the research carried out in these places. The following subcategories were found within the thematic research category: scientific dissemination, which includes news from scientific events; publisher; notices; and research projects.

During the period of data collection, we noticed that 31 articles in the FIG channel related in some way with the research category. Among them, the majority addressed issues related to institutional communication, such as the publication of calls for grants, events, or awards, without deepening the content of the research itself. Among the 31 articles, five had research projects as their main theme (Table 2). However, if the totality of the analyzed news is considered, this reverts to only 5% of the articles on scientific investigation in a period of four months. In the IF Goiano channel, 34 articles were published in the research category, but none of them portrayed research projects as their theme. The publications were mainly restricted to topics such as grants, internal promotion notices and disclosure of awards or events.

**Table 2:** Analysis of articles on research projects at one Institute.

Inclusion criteria	Journalistic material 1	Journalistic material 2	Journalistic material 3	Journalistic material 4	Journalistic material 5
**Knowledge area**	Chemistry	Sustainability	Environment	Innovation	Innovation Chemistry and Electronics
**Institutional nature**	Scientific research	Postgraduate studies	Postgraduate studies	Scientific research	Scientific research
**Nature of** **the sources**	Officers: ● Researchers ● Teachers and students	Officers: ● Teacher and student researchers ● Municipal government	Officers: ● Researcher professor ● Municipal government	Officers: ● Researchers ● Teacher and student researchers	● Researcher professors
**Previous knowledge**	Yes	No	Yes	No	Yes
**Language**	● Presence of technical terms that are difficult to understand ● Absence of glossary	● Easy to understand ● No glossary required	● Technical terms ● Absence of glossary	● Easy to understand	● Technical terms ● Absence of glossary
**Focus on journalism**	Yes	Yes	Yes	No	Yes
**Reporting criteria**	In parts	Most of them	In parts	In parts	Yes
**Content**	● Superficial ● Link to the article ● Restricted to source speech	● Contextualization ● No link to the survey ● Expansion beyond sources	● Small contextualization ● Medium deepening	● Superficial ● Survey information, but no link ● Restricted to source speech	● Medium deepening ● Restricted to information from sources
**Visual aspects**	Photographers of the researchers	Photographs and maps	Photos	Experiment photography	● Photograph of the experiment

Table 2 shows the aspects analyzed in relation to textual analysis according to the categories previously established: subjects and areas, nature of the sources consulted, need for prior knowledge, language, focus on science journalism or scientific dissemination, content, and service to news criteria (relevance, scope, timeliness, novelty).

The results in Table 2 show that most of the research dissemination work occurs within the scope of journalism, not necessarily scientific, but considering the criteria and characteristics of the news. Despite this, it still presents content essentially linked to official sources, disregarding a consolidation of the theme, facts, and characters. The presence of technical terms is also detected in three of the five subjects, without explanation through a scientific glossary or exemplification. The visual aspect is also limited to photographs by researchers or of experiments. Only one of them includes maps related to the study.

### c) Characterization of the research subjects

The interviews in this study were conducted with participants from all the groups (n = 30). In the CM group, the two manager have masters, one being a journalist and the other, public relations professionals with 7 and 8 years of work experience in the IF, respectively. Among the members of the JC group, three are female and two are male, with two having specialization and three having masters. Only one of them has a degree in literature, and the rest are graduated in social communication with a degree in journalism. Most are between 30 and 37 years old. Their work experience at the institution varies between 6 and 13 years.

The RD group was formed by two participants with a background in pedagogy and engineering, and 10 and 25 years of work experience, respectively. The academic backgrounds of the members of the RM group (n = 5, 100% male with doctorate) are in veterinary medicine, agricultural science, chemistry, industrial chemistry, and history. They are between 35 and 43 years old, and have 9 to 16 years of work experience in the IF.

Among the members of the ReS group (n = 8), five are male and three are female. They are between 33 and 53 years old and have between 5 to 13 years of work experience at the Institute, except one who has 30 years of experience at the institution. All have doctorate. The StuR group (n = 8) consists of four from each sex. They are between 20 and 27 years old and are in higher education between 2 and 4 years old. They are studying literature, information sciences, biological sciences, logistics, agronomy, food engineering, and social sciences.

Regarding the quantitative sample, a total of 242 subjects answered the questionnaire. JC group is constituted by 23 subjects, being 4.3% with higher education, 52.2% are specialists, and 43.5% have masters. In the RM group, among the subjects (n = 11), 9% are specialists, 45.5% have masters, and 45.5% have doctorate. Regarding the StuR group (n = 52), 17.3% have incomplete secondary education, 73.1% have incomplete higher education, 7.7% have higher education, and 1.9% are specialists. In the ReS group (n = 156), 1.3% have specialization, 25.6% of them have masters, and 73.1% have doctorate. For each group, sex percentage, age and years of experience in the Institute are presented in the Table 1.

### d) Perceptions, relationships, expectations, and challenges

The results of the analysis of the interviews were grouped into three thematic categories: perceptions regarding the work of scientific dissemination carried out by journalists and communicators, relationship between journalists and researchers, and challenges to science communication. The latter category includes expectations for overcoming these challenges. These thematic categories were analyzed according to the context units (CUs) below, in each group, except for the StuR group, which contributed only to CU 1:

### Context units (CUs)

1. Perceptions: How do you consider the work carried out by the communication sector of the unit or institution in relation to scientific dissemination and research in progress?
2. Relationship: How do you perceive the relationship between journalists and researchers, and how can this relationship contribute to improving disclosure?
3. Challenges: What are the challenges faced today in this process of disseminating research at the institution? Do you have suggestions and expectations on how to overcome them?

In each axis, the initial categories of analysis and specific indicators were elaborated in registration units (Supplementary material 2 in S2 File), for each group. For the RD group, we registered three categories: Reality, Evaluation, and Communication with society. For the RM group also, three categories were pointed out: Evaluation, Incipient Construction, and Reassignment. For the group of researchers (StuR and ReS), the following categories were listed: Incipient Disclosure, Positive Disclosure, Perspectives, Needs, Appointments, Internal Communication, and Communication with Society.

Regarding the evaluation of the RD (Supplementary Material 2 in S2 File) of the two institutions, they realize that scientific dissemination is still quite incipient. This means that the work is largely restricted to internal disclosure, focused on institutional and administrative actions. The focus is, therefore, on institutional themes and not necessarily on the dissemination of research. So, there is a broad path for the collective construction of these relationships. RDs also point out that the biggest challenge is in effectively achieving external reach of this scientific dissemination and improving institutional communication channels. This panorama is reported in the speech below, according to the category Communication with society (Supplementary Material 2 in S2 File):

“And another challenge is disclosure to society in general, right? Which I think is the role of communication. The researcher manages to reach and speak well perhaps to his peers, but he often has difficulty in speaking to the general population. There comes the role of the communicator to transform the scientific text, a technical text, into a more popular language.” (RD1)

The RMs’ views (Supplementary Material 2 in S2 File) are in line with that of the RD, expanding the perspectives along the three thematic axes. For them, in addition to consolidating external communication, the channels for disseminating research are insufficient for researchers and unattractive for people. Thus, according to the managers, external disclosure does not accompany the scientific production process of the institutions, and there is no planning for this work. In this regard, they also point out the need for improvement in communication and research flows, along with the construction of the relationship between journalists and researchers.

One of the main points in the entire process of scientific dissemination and science journalism, for RM group, is to encourage the proactivity of the two segments in favor of the science communication in these FIs. In addition to the actions, the RMs assess that the challenges involve changes in the culture of communication and the culture of researchers, giving new meaning to processes and work, including the journalist going to the field, their access to research information, and the establishment of a scientific dissemination agenda. This is reflected in the statements below:

“All of our research dissemination mechanisms are still insufficient. Our website is insufficient for the dissemination of research; it needs to be thought out and improved. We need to think about a strategy to disseminate research and even build a strategy, […] not only at the campus level, but also at the institutional level.” (RM5)

“Because when we, the researchers, create tools, it is because we are studying something. But, its application can happen in something else. So, the society will also use it […] if we keep everything to ourselves, we will not be able to affect society. I think we have improved a bit, but we must take further steps. I tell us because it is not only about communication, but also that communication must be articulated to those who are doing research, this Board here, for example.” (RM3)

In the same view, adding to the research subjects’ scores, ReS perceive the lack of relationship between the institutions and the press in each city where they have campuses (Supplementary material 2 in S2 File). However, one of the problems pointed out in this regard would be the lack of institutionalization of scientific dissemination. They also point out that it is necessary to break the barriers so that the relationship between journalists and researchers can flow:

“I think we need to improve a lot. This is a point that I think. With the political situation that we live in [Brazil], we need to put society in our defense. For us to achieve this, the society needs to know what we are doing and what has been researched.” (ReS 4)

“We need to break the barrier of researchers and scientists in order to leave, at times, a certain distance or even an aversion in relation to journalistic language, because this hinders the dissemination of scientific research and, at the same time, tries to bring communication professionals and journalists in the institution closer to what we do, because this distance often even causes ignorance about what happens in the institution.” (ReS 1)

“I think that researchers should be willing to get involved with this type of activity and try to make the most of it […] the journalist, who can assist in this process, also has to be available and interested in doing that. […] Perhaps, the question of institutionalization is interesting because it demonstrates that the institution is also interested in doing that. Once the institution is interested, the researcher and the journalist will also get interested.” (ReS 4)

The student researchers (StuR) also point out that the dissemination in general is positive and well performed, but that there are enough efforts from the communication sectors to publicize the research (Supplementary material 2 in S2 File). The students’ perspective is that these sectors get closer to the student groups, also establishing a dialogue with the students in favor of scientific dissemination. In addition, a journalist specifically can carry out these scientific guidelines periodically in the units. The perceptions are portrayed in the statements below:

“I believe that, in general, the communication part of the campus… is good, very good. Of the times that we carry out programming with educational activities, they have always been disclosed, broadcast. Now, with the scientific part, I do not feel much commitment. It is more in the disclosures for students to participate, but I do not see much being produced from the final product.” (StuR1)

“So, I believe that maybe a disclosure, I do not know, there is a way to bring the student closer, you know. For example, there is the guild; there are academic centers. So, communication gets closer to these vehicles […], with more ease, than just playing on the net, playing on the website, and waiting for someone to go there and access it.” (StuR8)

“I believe that in order to better convey the issue of both the initial product and research product, it would be necessary to have at least one journalist on the publicity side […] to stay specifically on the research part and come to the campus every week.” (StuR1)

The vision of the communication managers, journalists, and communicators (Supplementary material 3 in S3 File) mainly goes through the improvement of communication and research flows, with the availability of initial and final information on projects and ongoing research. This is depicted in the registration units below:

“I think there has to be an open system that would bring information including the title of that research, name and contact of the researchers, a summary of that research, the project itself, and also […] some reports are being produced; even reading these reports allows the journalist or communicator to know on what step he is. Also, there are hundreds of researches. I will not be able to call all of them to find out how it is. So, the system facilitates, and from what is fed into the system, arouses interest.” (CM2)

In addition, they also point out, as well as managers and researchers, the lack of planning, the priority for institutional communication actions and not the science communication, and work only at the provocation of researchers. To improve the relationship with researchers, they affirm the need for closer work by this group and proactivity on the part of journalists and scientists. Among the challenges, in addition to this relationship, are the institutionalization of scientific dissemination in the institutions, the development of joint work between sectors, the implementation of an open research system, a publishing agenda, and the creation of efficient and effective channels and products. These aspects are highlighted in the statements below:

“Sometimes, we are very involved in spreading the word about teaching and extension, and research… because there is more accurate work; it has to be done in the best possible way. But, I do not remember having the communication team discussing the issue of the implementation of scientific journalism, improvements, and the exchange of experiences in almost ten years. We do it as the demands arise. But, I see that there is an effort; we always see research being released at some point.” (JC4)

“I think that in addition to the researcher needing to become more aware that what he produces is notable, we also lack a dialogue with these researchers and students to know what they are producing. Often, the researcher seeks us to disseminate the research. There, due to various factors, the administrative routine and other types of communication—administrative and internal—which also take up our time, we have to program ourselves to make this scientific dissemination because it is different from other communications.” (JC2)

“There is an abyss today and until we manage to reach it, we have to go through many places. It is the Research Dean who will contact the research directorate; in short, there are many filters until we reach there. We do not even know if the surveys that come to us are the best to be released at the moment, if there is something we are not aware of. In short, it goes through too many filters until it reaches us. So, we establish an initiative to shorten this path […]” (JC1)

In the second stage of the analysis of the interviews, the final categories were established (Table 3) by converging the statements between all the groups. These results were obtained from the triangulation between the opinions of the groups and between the categories initially detected separately by segment.

**Table 3:** Final categorization by thematic axis and by groups.

Thematic axis/*Category*	Indicators	JC	R	
**Perceptions**				
*Communication with society*	Lack of external reach and relationship with the press			
*Institutional focus*	Prioritize science communication			
*Planning*	Create channels and fit the public			
	Lack of systematized actions			
*Barriers*	Mutual proactivity			
	On-demand service			
**Relationship**				
*Building relationships*	Articulate research and communication sectors			
	Integrate journalists and researchers			
	Need for improvement			
	Develop work with researchers			
	Good relationship with small groups			
*Collective effort*	Open dialogue on both sides			
	Promote accessibility to journalists			
	Promote accessibility to researchers			
	Approaching researcher and student groups			
	Breaking down relationship and language barriers			
*Attitude*	Mutual proactivity (researcher and journalist)			
**Challenges and expectations**				
*Reach*	Expand connection with society			
*Expectation of processes and planning*	Expand and improve processes, production, and channels			
	Establish disclosure agenda and routine			
	Create open system with search data			
	Mapping and selection of topics of public interest			
	Scientific dissemination in the field, contextualized, and continuous			
	New channels: diversification and multimedia			
	Training journalists and researchers			
*Flows*	Strengthening ties: researchers, communication, and the research sector			
	Access to researchers			
	Access to research information			
*Expectations in science communication*	Changing culture of science communication			
	Change of research culture			
	Investments in personnel and communication equipment			
*Cooperation*	Develop joint work			

JC (Journalist and communicator); R (supervisor and student researchers and research managers). The cell (s) filled with grey color means (s) that the category and indicator (s) was/were mentioned by the group (s) in question.

The results in Table 3 point to the convergence of perceptions in the three thematic axes in two large groups, and the opinions consolidated by these groups separately. The CMs were grouped with the JCs, as they all belong to the social communication area. Researchers and RM were also grouped together because they are all researchers. Given the above, the groups perceive the scenario of scientific dissemination in institutions differently in 15 indicators (Table 3)—in spaces where the color grey does not match in both columns—which will be complemented by data from the quantitative research in the next section.

### e) Scenario of scientific dissemination and the roles of each actor in this context

This section contains subsidies that complement the previous section and that match the analyzed responses, according to the groups participating in this research. Two thematic axes are addressed: Perceptions and Relationship (Table 4).

**Table 4:** Perceptions and relationship between communicators and researchers.

	JC	RM	StuR	ReS
	n(%)	n(%)	n(%)	n(%)
Scientific research carried out at the Institution is properly disclosed				
I disagree	5(21.8)	5(45.4)	9(17.3)	74(47.4)
Undecided	7(30.4)	3(27.3)	17(32.7)	44(28.2)
I agree	11(47.8)	3(27.3)	26(50)	38(24.4)
Total	23(100)	11(100)	52(100)	156(100)
Communication professionals interact with researchers				
I disagree	2(8.7)	7(63.6)	16(30.8)	74(47.4)
Undecided	13(56.5)	1(9.1)	20(38.4)	43(27.6)
I agree	8(34.8)	3(27.3)	16(30.8)	39(25)
Total	23(100)	11(100)	52(100)	156(100)
Communication professionals are accessible				
I disagree	1(4.3)	4(36.4)	9(17.3)	37(23.7)
Undecided	2(8.7)	2(18.2)	19(36.5)	35(22.4)
I agree	20(87)	5(45.4)	24(46.2)	84(53.9)
Total	23(100)	11(100)	52(100)	156(100)
Journalists and communicators are the only ones who can make scientific dissemination				
I disagree	13(56.5)	9(81.8)	27(51.9)	106(68)
Undecided	6(26.1)	2(18.2)	15(28.9)	29(18.6)
I agree	4(17.4)	0(0)	10(19.2)	21(13.4)
Total	23(100)	11(100)	52(100)	156(100)
Researchers can do scientific communication				
I disagree	0(0)	1(9.1)	2(3.8)	15(9.6)
Undecided	6(26.1)	1(9.1)	12(23.1)	33(21.2)
I agree	17(73.9)	9(81.8)	38(73.1)	108(69.2)
Total	23(100)	11(100)	52(100)	156(100)

*Note*. Results of the questionnaire according to the Likert scale.

JC (journalist and communicator); RM (research manager); StuR (student researcher); ReS (Research Supervisors).

Our results demonstrate that the groups perceive that scientific dissemination is not carried out properly in the institutions (Table 4). In each group, the scenario is unfavorable to the work of journalists and communicators in publicizing science. However, there are different views between the JC and StuR groups and RM and ReS. Opinions among them vary, converging to a negative assessment of scientific dissemination. Regarding the relationship between journalists and researchers, which in Table 4 is translated by the interaction between groups and accessibility of communication professionals, the evaluation of the research subjects is also not favorable. As for the accessibility of communication professionals, the JC group’s understanding is that they are accessible. In the RM group, most respondents either are undecided or disagree with the statement. Most students disagree with the statement or are undecided. ReS agree that communication professionals are accessible.

## Discussion

In the context of the qualitative and quantitative stages of this study, our aim was to identify and analyze the scientists’ perceptions regarding the work of journalists and communicators, the relationship between these groups, and the challenges of science communication. Thus, the data from different collection instruments provided an analysis of the aspects that surround the development of scientific dissemination in the investigated FIs. The three thematic axes presented—perceptions, relationships and challenges—were shown as an intertwining of connections that, together, try to map the reality of this dissemination, especially by the main actors who participate in the process: researchers, and journalists and communicators. This complexity of science communication may translate into these issues that hamper the communication process of research, since scientific dissemination is more complex than just translating science jargons into a language that the public understands. It is part of a historical and organizational chain; the roles of these actors and objectives have already been researched by Koivumäki & Wilkinson [20]. Its complexity stems from the diversity and interconnection of its various elements including the communication objectives, the content being communicated, the format in which it is presented, and the people and organizations involved [45].

These channels are also marked by intense user participation, as mentioned by López & Altamirano [46] while stating that we are in the “age of participation” and no longer in the age of information. According to this participatory model, called the interactive symmetry model, organizations need to seek allies in the development of strategies and “achieve balance so that these communicative processes take place in plans of equality and mutual influence, in which both have initiative in handling the their relationships, and individual or group response” [46].

Organizations and institutions where science is disseminated historically and socially contextualize their own concerns and influences. Further, the communication landscape is changing dramatically as they offer unprecedented opportunities to communicate and connect with others; but, they also pose many challenges [45,47]. A primary resource for these studies is to identify the key factors and best practices for effective science communication that anticipates and manages to deal with this complexity, just as organizations are complex.

We realize that the evaluation of the groups that scientific dissemination is insufficient in the institutions is not a reality restricted to the FIs, but has already been highlighted in research by Bueno [48,49]. The author not only mentions the so-called scientific “invisibility” in the digital communication of public institutions, but also the challenges for research to reach society. Moreover, organizations can communicate outside the walls, contributing to the public debate on science, technology, and innovation.

In this context, Massarani & Moreira [47] broaden the range of challenges, extrapolating from internal relations or the inserted work of the institutions to the effective contributions in basic science education. This is one of the premises of the IF, through their technical courses integrated to high school and humanistic education [50] present in institutional laws and regulations. Further, the authors point out the need for intense and qualified work to be carried out on social networks and mass media, and to bring science where people are. The aim is to promote actions that seek visibility to the importance of science for economic and social development, such that innovation and creativity stimulate technological and industrial growth in modern nations [15].

In the meantime, the opinions of researchers, and journalists and communicators evaluated in the present study converge on the need to expand the performance of the communication sectors with planning, changing the communication and scientific culture, developing actions, and building a dialogued relationship with researchers. Moreover, the analysis of journalistic articles is consistent with both the statements presented by the research subjects and the results of the quantitative research regarding the insufficiency of scientific dissemination in the two institutions. Among the news collected, it is possible to perceive a predominance in the two FIs, of matters about management, teaching, or extension, directed to internal character, which shows the speech of the groups when they reiterate that the focus of the communication work is institutional and attention is not paid to publicizing the research. With this, the external communication indicators presented by the research subjects are confirmed, pointing out the need to change the culture of communication—scientific and cultural [51,52]—and the elaboration of a science dissemination planning, thus promoting a dissemination agenda [8,9].

The gap in relations and the distance between scientists and communication advisors have already been addressed by Massarani & Peters [53]. Thus, the construction of a scientific culture in the contemporary world may be possible through a process of reflection of science itself, but by something that is not science. At the same time, it is also a constituent part of contemporary science and occurs through communication, more specifically, through science communication [51]. Other factors pointed out by the subjects of our research regarding the relationship between journalists and researchers corroborate the studies of Rose et al. [12]. They comment that even with the efforts of communication sectors in the practice of science communication, there are obstacles to this process such as the lack of support and a policy of scientific dissemination. This is also in line with the scores of the subjects in this study when mentioning institutionalization as an important step to plan and execute scientific dissemination.

There are Brazilian public institutions—mainly universities—that in the last two years have managed to advance in the creation of a culture of science communication or at least in the prioritization of scientific contents. An example is the Federal University of Uberlândia (Universidade Federal de Uberlândia—Brazil), which, in addition to initiating its activities in science communication and science journalism through the communication sector, has more recently managed to implant products. These contribute to the popularization of science and the fight against disinformation, and were triggered, mainly, due to the current moment of social isolation due to the Covid-19 pandemic [54].

It is important, therefore, to think about dialogue and building with multiple hands, with the meaning in which Silva and Machado [46] assign score when they describe an experience of continuing teacher education, which starts from scientific knowledge, and meetings and discussions between different groups in addition to the various active voices such as the foundations. They highlight “a job that is not defined a priori, but that values the construction process for many hands, or as it was said, a job ‘in the network, in the network and with the network,’ already pointing to a job ‘beyond from the Web.”

The sectors of social communication should to be recognized in this institutional environment for their ability to deepen in communication (science) issues. Thus, it is necessary to move forward with strategic (planning) purposes, which go beyond operational activities. This recognition is necessary among the communicators themselves and in other instances. Even if this collective condition of creation does not take effect at first, the institutional management and communication management could develop spaces and time in the administrative routine for joint debates. Still, it is important to practice dialogue that is so necessary in this process of integration between communication and research, and that seeks the realization of a truly collective science communication project, which meets the precepts of circulation of scientific information and democratization of knowledge.

The findings of this study are not representative of all FIs in the country since they have a geographical limitation, as they only refer to two FIs in central Brazil. Further, the research subjects are restricted to participants in scientific initiation programs and, therefore, are only part of the research context in the institutions. However, the research presents an overview of the relationships and perceptions between the main parties involved in the process of science communication in two institutions of the same teaching network and national science. Even with diversified characteristics and backgrounds, the two FIs showed similarity regarding the analysis of scientific dissemination in their institutional environments. Furthermore, the research brings interesting contributions regarding the methods by presenting different forms of data collection and the intersection between them, which is a characteristic of mixed research.

The scenario analysis of two institutions hardly occurs in research of this nature. There are few studies focusing on the organizational environment, especially those that analyze this change in focus and relationships in science communication practices [55]. Therefore, we believe that the results of our study can assist in the elaboration of strategies to strengthen the practice of science communication within these FIs. Our findings may contribute to future and expanded studies of science communication and science journalism in other Brazilian FIs.

## Final considerations

The main findings of this study indicate that researchers and research managers perceive that the dissemination of research is insufficient in relation to the scientific production routine of the analyzed IFs. For journalists, communicators, and communication managers, it is necessary to create a plan for scientific dissemination and awaken the teamwork between of researchers and communicators and allow them to develop their own initiative and independence. All groups consider that the institutionalization of scientific dissemination is fundamental in this process.

The relationship between journalists and researchers, which occur timidly in isolated movements on the institutions’ campuses, still need to be built. This construction also needs to focus on articulated work between research and communication sectors so that the main challenges are overcome. Among them are institutionalizing scientific dissemination, establishing a science communication plan, expanding its reach in society, and overcoming the institutional barriers to standardize scientific dissemination and the relationship between groups. Moreover, institutional documents should be re-discussed and reconstructed to properly consider science communication and science journalism in a continuous institutionalization process.

## Supporting information

S1 File Fully interview guide in Portuguese (original version) and English (translated version). (DOCX)

S2 File Initial thematic categorical analysis of the interviews with researchers, research dean, and research managers. (DOCX)

S3 File Initial thematic categorical analysis of the interviews with communication managers, journalists, and communicators. (DOCX)
