# Supplementary material for: Perceptions, relationships, expectations, and challenges: Views of communication and research for scientific dissemination in Brazilian Federal Institutes
Source: PLoS One. 2021 Oct 14;16(10):e0258492. doi: 10.1371/journal.pone.0258492 (PMC8516308; doi:10.1371/journal.pone.0258492)
Supplement: S1 File — (DOCX) [file pone.0258492.s001.docx]

**Supplementary material 1**

*Fully interview guide in Portuguese (original version) and English (translated version)*

| **Entrevista com jornalistas e comunicadores** | **Interview with journalists and communicators** |
| --- | --- |
| 1. Há quanto tempo atua em assessoria de comunicação?  2. Já teve experiência em veículos de comunicação? Por quanto tempo?  3. Você já teve algum contato profissional com o jornalismo científico? Se sim, conte um pouco como foi. Se não, imagina como ele ocorre, suas particularidades e finalidades?  4. Você exerce o jornalismo científico na sua prática profissional na Instituição? Se não, como acha possível que a prática do jornalismo científico ocorra na sua rotina diária de trabalho? Há espaço para isso?  5. Qual é a importância do jornalismo científico para a publicização das informações de ciência?  6. De que maneira você entende que o jornalismo científico de boa qualidade pode interferir para a transformação social na vida dos estudantes e da sociedade? E para dar visibilidade à instituição?  7. Qual é o seu papel como comunicador nesse contexto?  8. Como é o relacionamento entre jornalistas, comunicadores e cientistas na sua unidade?  9. Quais as expectativas em relação aos pontos que podem ser melhorados nessa relação?  10. Quais os desafios enfrentados hoje no processo de divulgação das pesquisas científicas na instituição? Como superá-los?  11. Qual é sua avaliação em relação ao que é feito na área de jornalismo científico/divulgação de pesquisas? Há uma publicação específica, canais e instrumentos para socialização da produção científica, no formato jornalístico e/ou no formato científico? Como as ações importantes são desenvolvidas?  12. Qual é a sua sugestão para se ampliar a divulgação jornalística de ciência no  Instituto, atrelando-a, também, às atividades de ensino e extensão, voltadas aos estudantes e à sociedade?  13. A estrutura de comunicação da instituição comporta diretrizes e iniciativas direcionadas à divulgação científica? Como isso poderia ser desenvolvido?  14. Como desenvolver um bom produto de comunicação para divulgação de pesquisas, quanto à forma, conteúdo, canal/canais de veiculação, dentre outros? Considere o público como sendo a sociedade em geral. | 1. How long have you been working in communication consultancy?  2. Have you had experience with communication media? For how long?  3. Have you had any professional contact with scientific journalism? If yes, tell us a little bit about how it was. If not, imagine how it occurs, its particularities, and purposes.  4. Do you practice scientific journalism in your professional practice at the institution? If not, how do you think it is possible for the practice of scientific journalism to occur in your daily routine of work? Is there room for that?  5. What is the importance of scientific journalism for the publication of scientific information?  6. What is your understanding of how good-quality scientific journalism can interfere with social transformation in the lives of students and society? Does it give visibility to the institution?  7. What is your role as a communicator in this context?  8. How is the relationship between journalists, communicators, and scientists in your unit?  9. What are the expectations regarding the points that can be improved on in this relationship?  10. What are the challenges faced today in the process of disseminating scientific research in the institution? How are they to be overcome?  11. What is your assessment of what is done in the field of scientific journalism/research dissemination? Is there a specific publication, and are there specific channels and instruments for the socialization of scientific production in journalistic and/or in scientific format? How are important actions developed?  12. How do you suggest expanding the journalistic dissemination of science in the Institute, also linking it to teaching and extension activities, aimed at students and society?  13. Does the institution's communication structure include guidelines and initiatives directed at scientific dissemination? How could this be developed?  14. How can a good communication product for research dissemination, as to the form, content, channel/broadcasting channels, among other categories, be developed? Consider the public as being society in general. |

| **Entrevista com gestores de Comunicação** | **Interview with Communication Managers** |
| --- | --- |
| 1. Qual é sua formação acadêmica?  2. Há quanto tempo exerce função de gestão na comunicação do Instituto?  3. Qual é a sua avaliação da atuação da comunicação institucional - Reitoria e campi - na divulgação das pesquisas científicas realizadas no Instituto?  4. O que justifica esse contexto?  5. Em quais aspectos ele pode melhorar para se ter uma divulgação científica eficiente e que seja atraente aos diversos públicos?  6. De que forma o jornalismo científico está presente no dia a dia das atividades do setor de comunicação?  7. Como você avalia a relação do setor de comunicação da Reitoria com os pesquisadores da Instituição?  8. Qual é o seu papel como profissional e o do setor no incentivo às atividades de publicização da ciência no Instituto?  9. De que maneira essa relação pode influenciar no seu trabalho, no que tange à divulgação científica?  10. Quais ações poderiam ser desenvolvidas para melhorar o fluxo de informações científicas que chegam ao setor e o acesso dos jornalistas aos cientistas da Instituição?  11. Como você avalia que a ampliação da divulgação das pesquisas pode contribuir para a Instituição, para os sujeitos envolvidos nos projetos e para a sociedade em geral?  12. Qual é a sua avaliação das contribuições da pesquisa científica na vida acadêmica, profissional e pessoal do estudante? | 1. What is your academic background?  2. How long have you had a management role in the Institute's communication?  3. What is your assessment of the performance of institutional communication—Rectory and campuses— in the dissemination of scientific research carried out at the Institute?  4. What justifies this context?  5. In what respects does it represent an improvement to have efficient and attractive scientific dissemination for different audiences?  6. How is scientific journalism present in the daily activities of the communication sector?  7. How do you evaluate the relationship of the Rectory's communication sector with the Institution researchers?  8. What is your role as a professional and that of the sector in encouraging activities publicizing science at the Institute?  9. How can this relationship influence your work, with regard to scientific divulgation?  10. What actions could be taken to improve the flow of information to scientists arriving in the sector and the access journalists have to scientists from the Institution?  11. What is your assessment of how the expanded dissemination of research can contribute to the Institution, for the subjects involved in the projects, and for society in general?  12. What is your assessment of the contributions of scientific research to academic life, professional and personal students? |

| **Entrevista com pró-reitores de Pesquisa e Pós-graduação** | **Interview with Deans of Research and Graduate** |
| --- | --- |
| 1. Qual é a sua área de formação?  2. Há quanto tempo exerce a função de pró-reitor? Já exerceu outro cargo de gestão na pesquisa?  3. Qual é a sua avaliação em relação ao desenvolvimento de pesquisas científicas no seu Instituto nos últimos dez anos?  4. Como você avalia a divulgação científica das pesquisas realizadas na Instituição antes de 2008? E a partir desse período até os dias atuais?  5. Qual é a importância do jornalismo científico nesse contexto institucional, ao levar o conhecimento científico ao grande público?  6. Como você avalia que a ampliação da divulgação das pesquisas pode contribuir para a Instituição, para os sujeitos envolvidos nos projetos e para a sociedade em geral?  7. Qual é a sua avaliação das contribuições da pesquisa científica na vida acadêmica, profissional e pessoal do estudante, principalmente o do ensino médio integrado e da graduação?  8. Como você percebe o relacionamento entre os pesquisadores e os jornalistas/comunicadores da instituição? Caso avalie que pode melhorar, tem sugestões?  9. Qual é a sua sugestão de produto educacional de comunicação para divulgação de pesquisas na Instituição? | 1. What is your area of training?  2. How long have you held the position of dean? Have you already held another management position in the search?  3. What is your assessment regarding the development of scientific research in your Institute in the last ten years?  4. How do you assess the scientific dissemination of research carried out at the Institution before 2008? And from that period to the present day?  5. What is the importance of scientific journalism in this institutional context, when taking scientific knowledge to the general public?  6. How do you assess the ability of expanded research dissemination to contribute to the Institution, to the subjects involved in the projects, and to society in general?  7. What is your assessment of the contributions of scientific research to academic life, students' professional and personal lives, mainly based on integrated high school and graduation?  8. How do you perceive the relationship between researchers and journalists/communicators from the institution? If you think you can improve, do you have  suggestions?  9. What is your suggestion for an educational communication product for dissemination of research at the institution? |

| **Roteiro de entrevista com gestores de Pesquisa** | **Interview with Research Managers** |
| --- | --- |
| 1. Há quanto tempo atua como gestor da área de Pesquisa?  2. Qual é a sua avaliação em relação ao cenário atual da pesquisa institucional na sua unidade/instituição?  3. Em quais aspectos essa realidade pode ser melhorada?  4. As pesquisas desenvolvidas refletem os princípios institucionais e estão atrelados à formação unitária do indivíduo?  5. No contexto escolar, qual é a importância da pesquisa para a formação integral dos estudantes?  6. Para o país, o que a pesquisa traz de contribuições para o crescimento e desenvolvimento nacionais?  7. Você acompanha as notícias veiculadas nos canais institucionais de divulgação, em especial as de divulgação de pesquisas ou temas de ciência?  8. Qual é a sua percepção do atual trabalho de comunicação/jornalismo realizado para publicação de produções científicas na sua unidade/instituição? E como era antes de 2008?  9. Quais são as suas expectativas em relação à atuação de um jornalista/comunicador especificamente para a área de divulgação científica? Levar em consideração os pilares institucionais.  10. Qual é o impacto da divulgação jornalística de ciência para as Instituições e para a sociedade?  11. Quais são os desafios para se comunicar mais e melhor as pesquisas desenvolvidas na sua unidade/instituição?  12. Como essa comunicação pode ser melhorada? Tanto interna como externamente?  13. Como você percebe que o relacionamento entre jornalistas e cientistas pode contribuir na melhoria da divulgação das pesquisas institucionais?  14. Como você considera que o trabalho de comunicação/jornalismo pode contribuir na perspectiva multidisciplinar com o ensino?  15. Como e por que tornar os assuntos de ciência mais atrativos ao público? | 1. How long have you worked as manager of the research area?  2. What is your assessment regarding the current scenario of institutional research in your unit/institution?  3. In what ways can this reality be improved?  4. Does the research developed reflect institutional principles, and is it linked to the unitary formation of the individual?  5. In the school context, what is the importance of research for the integral education of students?  6. For the country, what does the research brings in terms of contributions to growth and national development?  7. Do you follow the news broadcast on institutional dissemination channels, especially those for disseminating research or science topics?  8. What is your perception of the current communication/journalism work done for publication of scientific productions in your unit/institution? And how was it before 2008?  9. What are your expectations regarding the performance of a journalist/communicator specifically for the area of ​​scientific dissemination? Take into account the institutional pillars.  10. What is the impact of journalistic dissemination of science on institutions and on society?  11. What are the challenges to communicate more and better the research developed in your unit/institution?  12. How can this communication be improved? Both internally and externally?  13. How do you realize that the relationship between journalists and scientists can contribute to improving the dissemination of institutional research?  14. How do you consider that communication/journalism work can contribute to a multidisciplinary perspective with teaching?  15. How and why make science subjects more attractive to the public? |

| **Roteiro de entrevista com pesquisadores (orientadores)** | **Interview with researchers (supervisors)** |
| --- | --- |
| 1. Qual é a sua percepção do cenário atual da pesquisa na sua unidade/instituição, em termos de políticas institucionais, incentivos (não apenas financeiros), interesse dos estudantes e servidores em pesquisar, a inserção dela como princípio educativo na interação com o ensino e a extensão?  2. Como você acha que a sua unidade/Instituição valoriza a divulgação da pesquisa científica? Como melhorar?  3. Você percebe se os estudantes sabem a importância da pesquisa científica?  4. Existe algum trabalho de conscientização nesse sentido?  5. Qual(is) a(s) contribuição(ões) desses estudantes no processo da pesquisa científica?  6. Em que medida essas pesquisas podem contribuir para a vida desses estudantes fora do contexto escolar?  7. Como você considera o trabalho realizado pelo setor de comunicação da sua unidade e da Instituição em relação à divulgação científica/jornalismo científico?  8. Em quais aspectos ele pode melhorar?  9. Por que não se divulga mais as produções científicas na Instituição? Quais os melhores instrumentos e canais para que ela ocorra, de forma que desperte a atenção e gere engajamento?  10. Em relação à divulgação das pesquisas/resultados, como você avalia a importância da difusão do conhecimento científico em uma linguagem mais acessível ao público? Como um produto novo pode ser interessante, quanto à forma, linguagem, recursos, pensando no público externo?  11. Quais os desafios enfrentados na unidade/instituição para melhoria da divulgação científica? Como superá-los?  12. Na sua opinião, como é possível estabelecer um diálogo de qualidade entre jornalistas/comunicadores e pesquisadores/cientistas? | 1. What is your perception of the current research scenario in your unit/institution, in terms of institutional policies, incentives (not just financial), interest of the students and research supervisors, research insertion as an educational principle in interaction with teaching and extension?  2. How do you think your unit/institution values ​​the dissemination of the scientific research? How can it be improved?  3. Do you see if students know the importance of scientific research?  4. Is there any awareness work in this regard?  5. What are the contribution(s) of these students in the scientific research process?  6. To what extent does this research contribute to the lives of these students abroad in the school context?  7. How do you consider the work performed by your unit's communication sector and the institution in relation to scientific dissemination/scientific journalism?  8. In what ways could it improve?  9. Why is the institution's scientific production not better publicized? What are the better instruments and channels for it to occur, in a way that attracts attention and generates engagement?  10. Regarding the dissemination of research/results, how do you assess the importance of the dissemination of scientific knowledge in a language more accessible to the public? How can a new product be interesting, in terms of form, language, features, and thinking about the external audience?  11. What are the challenges faced in the unit/institution to improve scientific dissemination? How can they be overcome?  12. In your opinion, how is it possible to establish a quality dialogue between journalists/communicators and researchers/scientists? |

| **Roteiro de entrevista com pesquisadores estudantes** | **Interview with student researchers** |
| --- | --- |
| 1. Quando falamos em pesquisa e ciência, o que vem na sua cabeça? Soa como algo próximo do seu dia a dia ou distante?  2. Por que você acha que a pesquisa científica pode ser importante na sua formação intelectual, profissional e pessoal? Tanto na escola como no seu ambiente familiar, para o desenvolvimento da sua cidade, estado ou país?  3. Já atuou em algum dos programas de iniciação científica e tecnológica? Se sim, como foi?  4. Como você avalia as ações de pesquisa realizadas na sua unidade/instituição?  5. Você costuma ler jornais, revistas, mesmo as digitais? Quais? Com qual frequência?  6. Por que acha que estar informado dos mais variados assuntos pode ser importante, tanto na escola como para o seu dia a dia?  7. Quais os assuntos de maior interesse quando você busca informações fora dos livros?  8. Alguma vez já teve a curiosidade de ler em matérias de jornais ou em revistas científicas assuntos que tenham a ver com ciência? O que achou?  9. Se você já leu notícias e textos científicos sobre pesquisas, o que achou da linguagem utilizada e dos textos? Você tem sugestões?  10. O que você pensa quando se fala em divulgação científica? E em jornalismo científico, já ouviu essa expressão? Quais palavras ou frases vêm à sua cabeça?  11. Por que você acha que pode ser importante publicar informações sobre pesquisas científicas?  12. Como deve ser a linguagem desses textos, nos canais institucionais, para melhor entendimento das informações?  13. Quais os canais de comunicação da sua unidade/instituição que divulgam informações,  inclusive de ciência e pesquisa? Qual é o mais eficiente no diálogo com estudantes?  14. Com qual frequência costuma ler as notícias institucionais, seja no site, e-mail ou redes sociais, e notícias de pesquisas veiculadas nesses canais?  15. Como deve ser a linguagem desses textos, nos canais institucionais, para melhor entendimento das informações?  16. Como você considera o trabalho dos setores de comunicação da instituição, ou da coordenação de comunicação do seu campus, quanto à divulgação das pesquisas?  17. Como o setor de comunicação pode contribuir para ampliar a divulgação interna e externa das pesquisas, quais os instrumentos que mais atraem a atenção para essa finalidade? Você tem alguma sugestão? | 1. When we talk about research and science, what comes to your mind? Does it sound like something close to your daily life or far away?  2. Why do you think scientific research can be important in your intellectual, professional, and personal education? Both at school and in your family environment, for the development of your city, state, or country?  3. Have you worked in any of the scientific and technological initiation programs? If yes, how was it?  4. How do you evaluate the research actions carried out in your unit/institution?  5. Do you usually read newspapers and magazines, even digital ones? Which? How often?  6. Why do you think being informed of a wide range of subjects can be so important at school for your day-to-day?  7. What are the subjects of most interest when you are looking for information outside of books?  8. Have you ever had the curiosity to read articles in newspapers or magazines on scientific matters? What do you think?  9. If you've read news and scientific texts about research, what did you think of the language used and the texts? Do you have suggestions?  10. What do you think about when it comes to scientific dissemination? And in scientific journalism, have you ever heard that expression? What words or phrases come to mind?  11. Why do you think it might be important to publish scientific research information?  12. How should the language of these texts read, in institutional channels, for better understanding of the information?  13. What are the communication channels of your unit/institution that disseminate information, including science and research? What is the most effective in dialog with students?  14. How often do you read institutional news, whether on the web, email, or social networks, and research news broadcast on these channels?  15. How should the language of these texts read, in institutional channels, for better understanding of the information?  16. How do you consider the work of the communication sectors of the institution, or of the communication coordination on your campus, regarding the dissemination of research?  17. How can the communication sector contribute to expanding internal dissemination and external research, and which instruments attract the most attention to this goal? Do you have any suggestion? |
