# Supplementary material for: Perceptions, relationships, expectations, and challenges: Views of communication and research for scientific dissemination in Brazilian Federal Institutes
Source: PLoS One. 2021 Oct 14;16(10):e0258492. doi: 10.1371/journal.pone.0258492 (PMC8516308; doi:10.1371/journal.pone.0258492)
Supplement: S2 File — (DOCX) [file pone.0258492.s002.docx]

**Supplementary material 2**

*Initial thematic categorical analysis of the interviews with researchers, pro-rectors, and research managers*

| **Thematic axis** | **Category** | | | **Indicators** |
| --- | --- | --- | --- | --- |
| **RD** | | | | |
| Perceptions | Reality | | | - Communication performance is extremely broad  - Communication is inward  - Lack of disclosure to the external public  - Definition of institutional communication and management priorities  - Need for advancement: from general to specialized communication  - Expand communication to and with society |
| Relationship | Evaluation | | | - Incipient at institutional level  - Isolated cases on campuses  - There is room for advancement  - Articulation of joint work: communication and research sectors  - Ideal scenario: allocation of journalist in the research department of the rectory |
| Challenges | Communication with society | | | - External reach of scientific dissemination  - Improve communication channels  - Expanding product types: videos and proposing new actions |
| **RM** | | | | |
| Perceptions | | Evaluation | - Inefficiency of institutional channels for the researcher  - Focus on teaching and forgetting research  - Unattractive channels  - Lack of objectivity on the institutional website  - Intense focus on internal communication  - Lack of external communication  - External disclosure does not follow the results of the research  - Need for advancement for a “scientific social communication”  - There is effort on the part of the communication, but it is necessary to improve  - Expansion of operations in press vehicles  - Need for improvement in communication and research flows  - Lack of science communication planning | |
| Relationship | | Incipient construction | - Build this relationship between journalist and researcher  - Need for proactivity between the two segments  -Good accessibility of the two segments  - Structural issues undermine this relationship  - There is little involvement due to the institutional reality  - Lack of integration between communication and research | |
| Challenges | | Resignification | - Change in the culture of communication  - Culture change for researchers  - Establish dialogue between communication, research and management sectors  - Improvement of internal communication flows  - Create a specific channel  - Journalist needs to go to the field, to the labs  - Journalist access to information  - Increase contact with researchers  - Establish a dissemination agenda  - Connect the IF with society  - Invest in human resources  - Stimulate the researcher's proactivity | |
| Continuation  **Researchers** | | | | |
| Perceptions (ReS) | | Incipient disclosure | - External communication to be developed  - There is an effort to update the institutional website and social networks  - Lack of connection and insertion in the press  - Role of satisfactory internal communication  - Absence of dissemination of routine research | |
| Perceptions (StuR) | | Positive disclosure | - Disclosure of the research itself and its results on the website  - Publication of important event and other information | |
|  |  | Incipient disclosure | - Needs improvement  - There is no commitment to scientific dissemination  - Suggestion of disclosure of projects by video series  - Need external reach by the press | |
|  |  | Perspectives | - Communication needs to get closer to students: student groups  - Establishment of insertion opportunities in external media  - Availability of the communication professional periodic coverage on campus  - Creation of specific channel or location on websites | |
| **Research Supervisors** | | | | |
| Relationship | | Needs | - Institutionalization of scientific dissemination  - Institutional interest  - Connection through a research policy that values ​​scientific dissemination  - Closer ties needed  - Approaching and overcoming barriers  - Openness to dialogue | |
|  |  | Appointments | - Conducting periodic meetings between research and communication  - Production of interviews with researchers for sending to the press  - Creation of a research database to access information | |
| Challenges | | Internal communication | - Use of simple language  - Overcoming resistance by researchers to communicate in an accessible way  - Research prioritization  - Integration: teaching and research  - Integration: journalist and researcher  - Institutionalization of scientific dissemination  -Expansion: investments in communication personnel and equipment | |
|  |  | Communication with society | - Closer communication with society  - Greater reach in the external community for integration and return of research demand  - Creation of specific dissemination channels | |

Legend: RD (Research Dean - Pro-rectors of Research); RM (Research Managers); StuR (Researchers students); ReS (Research Supervisors).
