# Supplementary material for: Perceptions, relationships, expectations, and challenges: Views of communication and research for scientific dissemination in Brazilian Federal Institutes
Source: PLoS One. 2021 Oct 14;16(10):e0258492. doi: 10.1371/journal.pone.0258492 (PMC8516308; doi:10.1371/journal.pone.0258492)
Supplement: S3 File — (DOCX) [file pone.0258492.s003.docx]

**Supplementary material 3**

*Initial thematic categorical analysis of the interviews with communication managers, journalists, and communicators*

| **Thematic axis** | **Category** | **Indicators** |
| --- | --- | --- |
| **CM** | | |
| Perceptions | Reality | - Need for structural and flow improvement at all levels  - Sending information with appropriate language to the communication sectors  - Presence of technical terms in matters, at the request of the researchers  - Researchers' understanding of the language appropriate to the lay public  - Lack of systematization  - Lack of planning  - Punctual actions on demand  - Eventually present on some campuses |
| Relationship | Evaluation | - Needs improvement  - Important item in improving flows  - Excellent contact with a small group of researchers  - There is no diversity, groups that divulge are repeated  - Need to develop work with researchers  - Attempting to expand comes up against a limitation by the researcher in translating information |
| Challenges | Appointments | - Institutionalization of scientific dissemination  - Internal improvement of flows to reach the external community  - Improved access to researcher and information  - Proactive role of communication  - Proactive role of the researcher: interest in publicizing  - Joint work between the research and communication sectors |
|  | Actions | - Construction of disclosure as a historical process: start, progress and results  - Open system with survey data  - Mapping and selection of topics and research of public interest, carried out by the research sectors  - Systematized channel for receiving information, periodicity and planning  - Development of something more visually attractive to arouse interest |
| **JC** | | |
| Perceptions | Disarticulation | - Assistance on demand and at the provocation of the researcher  - Priority for institutional communication [and not specialized]  - Sporadic subjects from research projects  - Lack of partnership with other scientific dissemination actions  - There are no structured actions  - One-off initiatives  - Lack of publicity  - Lack of relationship with the press  - Need for greater incentive  - There is no planning  - Prioritizing the dissemination of events and not research and science |
| Relationship | Evaluation | - Space by provocation  - Good relationship, but needs to improve  - When there is provocation by communication, the result is satisfactory  - Researchers need to be proactive in passing on information |
| Challenges | Expectations | - Creation of a science dissemination culture  - Proactivity of communication in proposing initiatives and contact with the researcher  - Establishment of science dissemination routines on existing channels periodically  - Expansion of disclosure will stimulate new researchers |
|  | Propositions | - New channels: platform, news agency  - Training of researchers to understand the roles: journalist and researcher  - Training of journalists and communicators  - Diversification of media and channels: videos, podcasts etc.  - Creation of scientific sources database |
|  | Institutional priority | - Change of communication culture, starting with the area  - Prioritize science communication  - Strengthen ties with researchers  - Propose structured actions with a schedule and new tools  -Establish planning and insert scientific journalism in the communication plan  - Make science communication a priority in communication routines  - Dealing with small teams |
| Perceptions | Reality | - Need for structural and flow improvement at all levels  - Sending information with appropriate language to the communication sectors  - Presence of technical terms in matters, at the request of the researchers  - Researchers' understanding of the language appropriate to the lay public  - Lack of systematization  - Lack of planning  - Punctual actions on demand  - Eventually present on some campuses |
| Relationship | Evaluation | - Needs improvement  - Important item in improving flows  - Excellent contact with a small group of researchers  - There is no diversity, groups that divulge are repeated  - Need to develop work with researchers  - Attempting to expand comes up against a limitation by the researcher in translating information |
| Challenges | Appointments | - Institutionalization of science communication  - Internal improvement of flows to reach the external community  - Improved access to researcher and information  - Proactive role of communication  - Proactive role of the researcher: interest in publicizing  - Joint work between the research and communication sectors |
|  | Actions | - Construction of disclosure as a historical process: start, progress and results  - Open system with survey data  - Mapping and selection of topics and research of public interest, carried out by the research sectors  - Systematized channel for receiving information, periodicity, and planning  - Development of something more visually attractive to arouse interest |
| **Journalist / Communicators** | | |
| Perceptions | Disarticulation | - Assistance on demand and at the provocation of the researcher  - Priority for institutional communication [and not specialized]  - Sporadic subjects from research projects  - Lack of partnership with other scientific dissemination actions  - There are no structured actions  - One-off initiatives  - Lack of publicity  - Lack of relationship with the press  - Need for greater incentive  - There is no planning  - Prioritizing the dissemination of events and not research and science |
| Relationship | Evaluation | - Space by provocation  - Good relationship, but needs to improve  - When there is provocation by communication, the result is satisfactory  - Researchers need to be proactive in passing on information |
| Challenges | Expectations | - Creation of a science communication  - Proactivity of communication in proposing initiatives and contact with the researcher  - Establishment of science dissemination routines on existing channels periodically  - Expansion of disclosure will stimulate new researchers |
|  | Propositions | - New channels: platform, news agency  - Training of researchers to understand the roles: journalist and researcher  - Training of journalists and communicators  - Diversification of media and channels: videos, podcasts etc.  - Creation of scientific sources database |
|  | Institutional priority | - Change of communication culture, starting with the area  - Prioritize science communication  - Strengthen ties with researchers  - Propose structured actions with a schedule and new tools  - Establish planning and insert scientific journalism in the communication plan  - Make science communication a priority in communication routines  - Dealing with small teams |

Legend: CM (Communication Managers); JC (journalists / communicators)
